# Amniotic Fluid Embolism After Cervical Ripening

**DOI:** 10.7759/cureus.75212

**Published:** 2024-12-06

**Authors:** Madison French, Teresa Bernardes, Christine C Greves, Shannon Shellhammer, Steve Carlan

**Affiliations:** 1 Obstetrics, Orlando Regional Medical Center, Orlando, USA; 2 Internal Medicine, Orlando Regional Medical Center, Orlando, USA

**Keywords:** amniotic fluid embolism pathophysiology, amniotic fluid embolism treatment, cesarean birth, fetal outcome, pre-induction cervical ripening

## Abstract

Amniotic fluid embolism (AFE) is a rare condition that can have catastrophic maternal and infant consequences. It can lead to rapid multisystem failure and is responsible for a significant portion of maternal deaths. The diagnosis is frequently made late in the pathological process, and the treatment is mainly supportive and infant delivery. It cannot be prevented. Whether cervical ripening and labor induction are risk factors is controversial.

A 31-year-old woman who was undergoing cervical ripening and induction of labor at 38 weeks gestation for medication-controlled gestational diabetes (A2GDM) was admitted for delivery. She received five doses of 25 µg vaginal misoprostol serially and, ultimately, a Foley catheter cervical balloon. After approximately 24 hours, she had the sudden onset of unexpected persistent fetal bradycardia. Her cervix was 4 cm at the time of the fetal distress. When she arrived in the operating room, she was hypoxic and difficult to awaken. An emergency cesarean delivery was performed under general endotracheal anesthesia. Immediately after the delivery of a profoundly depressed and acidotic infant with an umbilical cord pH of 6.84, she became hypotensive, requiring vasopressors. After diffuse intravascular coagulation was diagnosed, treatment for AFE was implemented.

AFE has a high mortality rate, and the length of time needed to identify the condition and the availability of specialty resources are two elements that can affect the outcome. Newer alternative treatments, such as the supportive “A-OK” (atropine, ondansetron, and ketorolac administration) protocol for AFE, are discussed. Ultimately, both mother and baby survived and, at the six-month check, are doing well with no sequelae.

## Introduction

Amniotic fluid embolism (AFE) is an extremely rare obstetric condition that often has catastrophic outcomes. AFE occurs anywhere from 1.9 to 6.1 of 100,000 deliveries [1]. Maternal mortality ranges from 10% to 90% and accounts for 10% of all maternal deaths in developed countries [2,3]. Theoretically, entry of amniotic fluid, which contains foreign antigens, including the fetal cells, into the maternal circulation leads to a sudden activation of the maternal humoral and immunological system, resulting in a cytokine storm and the release of procoagulant substances and vasoactive chemicals. This is similar to the events in a systemic inflammatory response syndrome [4,5]. Acute pulmonary hypertension follows, and a severe ventilation-perfusion mismatch occurs along with a progressive cardiogenic pulmonary edema, which ultimately leads to maternal hypoxemic respiratory failure. In addition, the concurrent surge of intravascular inflammatory mediators can lead to disseminated intravascular coagulation (DIC) because of the triggering of the coagulation cascade [4]. The result is that without immediate support and active treatment, multisystem organ dysfunction and possibly failure ensue. This case is important to study because the patient had 24 hours of misoprostol cervical ripening followed by mechanical ripening before the AFE occurred. The event occurred in real-time in a resource-rich tertiary-care maternity unit, and the delivery happened in minutes. The neonate was profoundly depressed and acidotic. To our knowledge, this is the first case of AFE following the combined use of both pharmacological and mechanical methods of cervical ripening and labor induction. As such, the literature has been reviewed for cases of AFE associated with ripening.

## Case presentation

A 31-year-old G2P1001 was admitted in October 2023 to the maternity ward at a tertiary referral hospital at 38 weeks gestation for induction of labor due to medication-controlled gestational diabetes (A2GDM). She had no other significant medical, family, social, or surgical history. The current pregnancy was complicated by A2GDM, which was well controlled on diet and insulin detemir 12 units every night. Her obstetric history included a previous vaginal delivery with an outlet vacuum assist due to non-reassuring fetal status in late labor.

Upon admission, her vital signs were normal, and she was experiencing no pain or contractions. Her cervical examination revealed an unripe cervix, which was not favorable for induction. The cervix was thick, posterior, and closed. She was given one 25 µg dose of misoprostol vaginally to start the cervical ripening process. She received three additional 25 µg doses of misoprostol vaginally, spaced out by roughly four to five hours. Throughout this process, the fetal heart rate tracing remained reassuring, with a Category I strip and no sustained contraction pattern. Each cervical check showed the cervix remained closed, thick, and posterior. The patient only reported mild cramping before the fourth misoprostol dose.

The following day, her cervix was 1 centimeter dilated, and she was counseled and consented to the placement of a Foley balloon with 60 mL of fluid for mechanical cervical ripening. A fifth 25 µg dose of misoprostol was administered vaginally at this time as well. The patient was given 50 µg of intravenous (IV) fentanyl 20 minutes after placement for discomfort. The fetal heart rate tracing remained reassuring at Category I. An hour later, she required an additional 100 µg of IV fentanyl for pain relief. She remained afebrile throughout the process.

About 2.5 hours after the placement of the Foley balloon (or around 24 hours after admission), the patient experienced a spontaneous rupture of membranes and increased pain. Four minutes later, prolonged fetal heart deceleration was observed, and the Foley balloon was removed. At this point, her cervical exam revealed 4 cm dilation, 80% effacement, and the fetal head at -3 station. Uterine contractions were occurring every one to two minutes. Due to the low fetal heart tones, terbutaline 0.25 mg IV was administered as a tocolytic, and the patient was repositioned. Fetal heart tones remained in the 60's, and the decision was made to proceed with an emergency cesarean delivery.

She reported contraction pain, nausea, and vomiting once en route to the operating room (OR). Upon arrival in the OR, the patient was delirious, and anesthesia had difficulty arousing her. Her oxygen saturation was in the 80's, so she was quickly intubated and placed under general anesthesia. Within 90 seconds, the fetus was delivered with Apgar scores of 1 and 5 at one minute and five minutes, respectively. The infant was profoundly depressed with an umbilical cord gas of 6.84 (7.34 ± 0.07) and a base deficit of -17.1 mmol/l (3.0 ± 2.7). The placenta was delivered without signs of abruption.

Five minutes after delivery, the anesthesiologist notified the surgical team of persistent ST segment depression and hypotension, requiring IV pressors to increase the patient’s blood pressure. The case proceeded routinely with the addition of tranexamic acid (TXA). After the uterine incision was closed, significant oozing of blood was noted from all tissues, with minimal uterine atony. Given the high likelihood of DIC, it was discussed that the patient likely had an AFE, and the embolus probably occurred immediately before arriving in the OR.

The anesthesia team quickly drew stat labs and began initial treatment with the “A-OK” protocol, which consists of 0.2 mg atropine, 8 mg ondansetron, and 15 mg ketorolac, all given as IV pushes. The atropine was withheld due to persistent tachycardia. Blood products were ordered, and due to persistent heavy vaginal bleeding, a Bakri balloon was placed in the uterus. The patient received 5 u of packed red blood cells (pRBC), 6 u of cryoprecipitate, 4 u of fresh frozen plasma (FFP), and 2 u of platelets during the procedure.

The patient was transferred out of the OR, intubated, on IV pressors, and transferred to the intensive care unit (ICU). She was ultimately transferred from the maternity ICU to the adult main hospital ICU, where she underwent interventional radiology (IR) embolization of bilateral uterine arteries. During the embolization, she received 2 u pRBC and 1 u FFP. Serial labs were obtained to follow her progress (Table 1).

**Table 1:** Serial labs Serial labs detailing important variables, starting with admission NO: not obtained, GET: general endotracheal anesthesia, NGTD: no growth to date, ESBL: extended-spectrum beta-lactamase, ICU: intensive care unit

Clinical findings	On admission	First labs in OR	Day 1 ICU	Day 2 ICU	Day 3 ICU	Day 4	Day 13
Hemoglobin (g/dL)	11.5	7.3	9.1	7.7	7.5	7.4	10.8
Platelet (x10^9^/L)	217	146	81	74	96	106	538
Fibrinogen (milligrams per deciliter (mg/dL)	NO	57	151	399	NO	NO	NO
Creatinine (milligrams per deciliter (mg/dL)	0.8	0.87	0.96	0.61	0.56	0.54	0.6
BUN/Cr ratio (blood urea nitrogen/creatinine)	12.2	11.5	18.8	18	16.1	29.6	13.3
Arterial pH	-	7.23	7.35	NO	NO	NO	NO
Lactate millimoles per liter (mmol/L)	NO	NO	6.6	1.2	NO	NO	NO
Glucose mg/dL (milligrams per deciliter)	111	114	118	110	91	155	109
Blood culture	NO	NO	NO	ESBL, *E. coli* bacteremia	NGTD	NGTD	NGTD
Systolic blood pressure (mmHg)	102	80	105	115	120	128	125
Oxygen saturation (%)	100	95 (GET)	100 (intubated)	94 (extubated)	98	99	99

**Figure 1:**
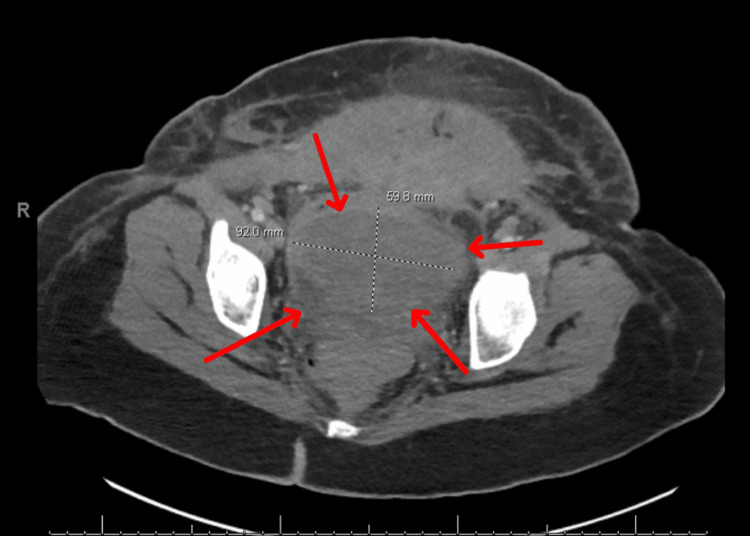
CT abdomen and pelvis. There is a 9 x 6 cm hematoma (red arrows) superior to the bladder. CT: computed tomography

She was extubated on postoperative day two, and the Bakari was removed. She developed a fever on postoperative day two and, over the course of four days, was diagnosed with extended-spectrum beta-lactamase (ESBL) *E. coli* bacteremia, fungal urinary tract infection, and anaerobic bacteremia and was started on the appropriate antibiotic and antifungal regimen. On postoperative day 8, there was a concern for expanding hematoma versus abdominal abscess on computed tomography (CT) imaging (Figure 1).

IR-guided drainage confirmed the mass was a hematoma. She continued to clinically improve and was discharged home on postoperative day 12. The neonate was discharged home in the first week of life with no signs of neurologic dysfunction. The patient and her newborn were followed by her ambulatory obstetric and pediatric team, respectively, for routine care without complications. She recovered uneventfully, and the infant met all of its milestones. In April of 2024, six months post-AFE, she had her last contact with our team. At that time, she and her infant were progressing normally, and she had returned to her pre-pregnancy health.

## Discussion

There are two important components that, in combination, make this case unique. First is the AFE that caused the immediate and devastating cardiorespiratory collapse that occurred in real-time in a high-resource hospital that had every possible advantage to save the mother and infant. Despite the immediate response, the infant was born acidotic, and the mother developed multisystem compromise. Second, the ripening process was lengthy and used five misoprostol placements and a Foley bulb placement. Whether this process of cervical ripening is a risk for AFE is controversial.

In developed nations, the mortality rate for AFE is highly variable and significantly related to the recognition rate of the condition and availability of emergency support services. In 90% of cases, the clinical presentation of AFE is abrupt, catastrophic, and rapidly progressive. Four criteria must be present to diagnose an AFE: (1) sudden cardiopulmonary arrest or systemic hypotension and evidence of respiratory compromise; (2) presence of DIC; (3) clinical evidence occurring during labor or within 30 minutes of placental delivery; and (4) no fever (≥38°C) during labor [6]. Common clinical manifestations include nausea, vomiting, anxiety, a sudden sense of doom with delirium, or altered mental status [7]. Sudden cardiopulmonary arrest, seizures, and hemorrhage can also be presenting features [8]. Other possible rapidly progressing pathologic processes, including umbilical cord prolapse, can also cause prolonged fetal bradycardia. In our case, after the cervical exam confirmed there was not a cord presenting, the next concern was for placental abruption. Given that the fetal status was not improving with maternal position changes and tocolytic administration, the only solution was for emergent delivery of the fetus. Once in the OR, she was hypoxic and difficult to awaken. The differential at that time included pulmonary embolism. Once anesthesia pointed out signs of cardiopulmonary strain and the patient was requiring pressors, there was concern about myocardial infarction. It was not until the patient presented with signs of DIC, combined with the overall clinical picture of pre-anesthesia confusion, desaturation, and ST wave depression that it was clear she likely had an AFE. Not only did the clinical picture fit the diagnosis of AFE, but the patient’s lab results that returned during the surgery and after confirmed the presence of DIC.

Treatment of AFE is difficult because multiple organ systems are malfunctioning simultaneously. It requires a multifocal, team-based approach involving the obstetric, anesthesia, critical care, nursing, and respiratory teams. Often, patients with an AFE are present already in cardiopulmonary arrest, and the first step in treatment is CPR [9]. However, in our patient’s case, the need for an emergency cesarean delivery may have been life-saving because, during the anesthesia process, the rapid recognition of hypotension and cardiac findings allowed for expedited hemodynamic support. Delivery of the fetus may improve outcomes [10]. In addition to delivery, appropriate fluid resuscitation, avoiding overload, cardiovascular support, and administration of blood products are the standard treatments for AFE. Norepinephrine is used to maintain blood pressure. Milrinone and dobutamine can be used to improve right ventricular contractility [11]. Typically, the massive transfusion protocol is initiated with a 1:1:1 ratio of pRBCs, platelets, and FFP [12]. TXA, a medication used routinely in obstetrics to not only treat but prevent postpartum hemorrhage, was used in our patient [13]. The "A-OK" protocol [14] consists of 0.2 mg atropine, 8 mg ondansetron, and 15 mg ketorolac, all given as IV pushes. It has been proposed that atropine and ondansetron may act to block serotonin and vagal stimulation, improving cardiovascular function. Additionally, rather than replacing consumed coagulation factors caused by the suspected coagulopathy, ketorolac inhibits thromboxane formation [14]. The timely administration of blood products was crucial to the immediate survival of this patient. The survival of both the mother and baby in this particular case is likely due to the prompt recognition and readily available emergency resources.

With regards to the cervical ripening and labor induction on the risks of AFE, there are conflicting reports. Several studies suggested that there might be a link [15,16]. However, more recent reports do not support an association of AFE with labor induction [17,18]. One paper specifically cites misoprostol use as a risk factor for AFE [19], but this finding is isolated and not confirmed by other research. The role of the length of time of ripening and induction has not been independently studied as a separate risk factor for AFE. In addition, the effect of pharmacologic ripening followed by mechanical ripening on the risk of AFE has not been studied or reported. One finding that is consistently reported to be a risk factor for AFE is ruptured membranes [20], which occurred spontaneously minutes before the AFE in our patient. Membrane rupture occurs in virtually all cases before delivery, so the specific variable that results in increased mixing of fetal substance with the maternal circulation in particular cases of AFE is not defined. Theoretically, the tachysystole of the accumulated ripening agents followed by membrane rupture is operative as a risk factor, but this cannot be confirmed and is only anecdotal in this case.

## Conclusions

While AFEs are extremely rare, and many obstetric providers will likely never see them in practice, it is necessary to understand the clinical manifestations of the calamity. It is important to always include the diagnosis as a differential when there is a concern for cardiopulmonary arrest in a mother in the labor, delivery, and immediate postpartum period. Additionally, this case clearly indicated that it should also be considered a differential diagnosis when there is a fetal compromise of unknown origin. Further, although the "A-OK" protocol is not an official treatment of AFE, it has been proven in several cases, and likely in this case as well, to decrease complications of the condition. This case highlighted the significance of how prompt recognition and action for both maternal and fetal status can be the rate-limiting step in the survival of AFE. Whether a combination of pharmacological and mechanical agents used over a prolonged time period is a risk factor for AFE is not known, but it is possible that the effect of excessive uterine contractions is an operative variable.
